# Full length transcriptomic profiling reveals insights into the white coat phenotype in Waardenburg syndrome mice harboring the *Mitf* R324del mutation

**DOI:** 10.1038/s41598-025-13359-8

**Published:** 2025-07-31

**Authors:** Wei Gong, Lu Ma, Zhili Feng, Xiangyao Zeng, Lile Ouyang, Yihan Hu, Xianlin Liu, Jie Wen, Xiaoming Kang, Yalan Liu, Hong Wu, Qiancheng Jing, Chufeng He, Yong Feng

**Affiliations:** 1 Department of Otorhinolaryngology, The Affiliated Changsha Central Hospital, Hengyang Medical School, University of South China, Changsha, China; 2 Institute of Otorhinolaryngology, Head and Neck Surgery, University of South China, Changsha, China; 3 MOE Key Lab of Rare Pediatric Diseases & Institute for Future Sciences, University of South China, Changsha, China; 4 Institute of Cytology and Genetics, Hengyang Medical School, University of South China, Hengyang, China; 5 Department of Otolaryngology-Head and Neck Surgery, Xiangya Hospital, Central South University, Changsha, China

**Keywords:** Full-length transcriptome sequencing, Waardenburg syndrome, *Mitf*, Pigmentation, Skin diseases, Gene expression

## Abstract

Waardenburg syndrome (WS) is distinguished by depigmented patches of hair and skin, striking blue eyes and sensorineural hearing loss. Studies on alternative splicing (AS) in the abnormal pigmentation in skin in WS are currently poorly understood. In this study, we conducted comprehensive full-length transcriptome sequencing analysis on abdominal skin tissues from mice harboring the *Mitf* p.R324del mutation and wild-type controls. Our analysis revealed 3619 differentially expressed genes (DEGs), with 1916 upregulated and 1703 downregulated. Notably, pigment-associated genes and related signaling pathways were significantly enriched. Additionally, we identified extensive AS events in the skin tissue of *Mitf* mutant mice, including 97 A3, 85 A5, 101 AF, 42 AL, 8 MX, 44 RI, and 184 SE events (*P* < 0.05), suggesting AS events may contribute to the pigment distribution patterns in Mitf p.R324del mutation mice. Furthermore, we observed downregulation of *Dct*, *Mlana*, and *Snai2*, which indicates that *Mitf* mutations disrupt neural crest cells into melanocytes, melanosome structure, and melanin biosynthesis. Overall, our results support that variation in gene expression and AS are important and complementary mechanisms governing pigmentation defects in WS.

**Supplementary Information:**

The online version contains supplementary material available at 10.1038/s41598-025-13359-8.

## Introduction

Waardenburg syndrome (WS), also known as auditory-pigmentary syndrome, is associated with various genes, such as *MITF*, *PAX3*, *SNAI2*, *SOX10*, *EDNRB*, and *EDN3*^1^. Waardenburg syndrome type 2 A (WS2A) predominantly manifests through abnormal skin and hair pigmentation, accompanied by sensorineural hearing loss^2^. The *MITF* gene is a major causative factor for WS2A, playing a pivotal role in melanocyte development, differentiation, survival, synthesis, and melanin transport^3^. Researchers have discovered over 100 mutations and variant sites within *MITF*, which comprises 17 exons, including nine exon 1 variants and 8 common exons^4^. The variability in exon 1 length leads to the existence of different *MITF* isoforms, with the shortest, M-*MITF*, being uniquely expressed in melanocytes. Extensive studies have indicated that the *MITF* gene is crucial for inner ear and melanocyte development^5^.

Melanocytes, derived from neural crest cells, undergo processes such as proliferation, migration, survival, and differentiation to become precursor cells, which eventually mature into melanocytes and terminally differentiate in regions like the skin, inner ear, and eye^6^. *MITF* regulates key genes involved in melanin synthesis, melanosome biogenesis, and melanocyte survival, making it a master regulator in pigmentation^7^. Given its high conservation across species, mouse models with *Mitf* mutations have been invaluable for studying the molecular mechanisms underlying WS and related pigmentary disorders. For instance, *Mitf* mutant mice exhibit coat color abnormalities and hearing deficits, closely mimicking the human WS phenotype^8^.

Full-length transcriptome sequencing, an advanced high-throughput technique, enables precise identification and quantification of isoforms, homologous genes, supergene families, and allelic gene expression transcripts, addressing limitations that previously hindered a comprehensive understanding of transcriptomics in biological processes^9^. Oxford Nanopore Technologies (ONT) has revolutionized this field with its long-read RNA sequencing technology, overcoming many of these previous constraints. This technology facilitates the analysis of variable splicing patterns, gene fusions, and the discovery of novel isoforms at the transcriptome level^10^. Alternative splicing (AS), a critical post-transcriptional process in mRNA, involves the selection of various exons during precursor RNA splicing, leading to the production of diverse mRNAs and, subsequently, a vast array of proteins. This diversity is indispensable for the variability observed in biological traits^11^. Given that over 90% of human genes undergo alternative splicing, it stands as a fundamental mechanism in pre-mRNA editing^12^. This process generates multiple mRNA isoforms from a single pre-mRNA, profoundly influencing transcriptome diversity and gene expression regulation^13^. ONT’s full-length sequencing technology adeptly detects AS events, thereby enhancing our capacity to analyze post-transcriptional AS occurrences^14^.

To gain a deeper understanding of the abnormal pigmentation caused by *Mitf* mutations, we successfully created a mouse model featuring the *Mitf* p.R324del mutation via CRISPR/Cas9 gene editing technology, which mimics the human *MITF* p.R217del mutation associated with WS2A^15^. Our model accurately replicated the human disease phenotype, with homozygous mutant mice exhibiting a pure white coat and hearing loss (unpublished data).

Building upon this success, we embarked on a comprehensive full-length transcriptome analysis of the skin tissues from *Mitf* p.R324del mutation mice and wild-type controls. Our study aimed to identify differentially expressed genes (DEGs) and AS events associated with the abnormal pigmentation observed in these mice. By integrating functional enrichment analyses and AS profiling, we sought to uncover the changes disrupted by the *Mitf* mutation, providing insights into the pathogenesis of WS and the role of *MITF* in melanocyte biology. This study not only advances our understanding of the molecular basis of WS but also highlight the potential of long-read transcriptomics in dissecting complex genetic disorders. By identifying key genes and splicing events involved in pigmentary abnormalities, our findings pave the way for future therapeutic strategies targeting MITF-related pathways.

## Materials and methods

### Sample collection, RNA extraction and quality control

In our study, we meticulously reared and genotyped offspring mice until they reached the weaning stage. Based on their genotypes, we divided the mice into two distinct groups: a homozygous group exhibiting a white coat and a wild-type group exhibiting a black coat, with each group consisting of three male mice. Following shaving, we collected skin tissues from the abdominal regions of each group. Total RNA extraction was carried out using the Total RNA Kit I (Omega, USA), and the quality of the extracted samples was rigorously assessed using a NanoDrop One spectrophotometer (NanoDrop Technologies, Wilmington, DE), a Qubit 3.0 Fluorometer (Life Technologies, Carlsbad, CA, USA), as well as through agarose gel electrophoresis. The genetically modified mice utilized in this study were generated and provided by Shanghai Model Organisms Center, Inc. and the study is reported in accordance with ARRIVE guidelines.

### Library construction and nanopore sequencing

After isolating approximately 500 ng of high-quality total RNA, we embarked on a series of procedures to prepare the sequencing library. These steps included reverse transcription, amplification, purification using AMPure beads (magnetic bead purification), adapter ligation for sequencing, and the preparation of the final library. The resulting cDNA library was then sequenced by the PromethION sequencer (Oxford Nanopore Technologies, Oxford, UK).

### Preprocessing of sequencing data

The raw data obtained from Nanopore sequencing are in the fast5 format, which encapsulates all the original sequencing signals. We used GUPPY (version 5.0.16) to convert these fast5 data into fastq format, capturing both the base sequence and its corresponding quality scores for subsequent analysis. To ensure high-quality reads, we filtered out sequences with quality scores below 7 and shorter than 50 bp, as these could include low-quality and adapter sequences that might hinder further analyses. This step is crucial for the reliability of subsequent information analysis results. For alignment, we utilized minimap2 (version 2.17-r941) to map the filtered reads against the mouse reference genome (ENSEMBL 109). Following this, we employed Pinfish (version 0.1.0) to construct a non-redundant transcriptome from full-length sequences. This transcriptome was then refined through clustering, deduplication, and correction processes to produce a set of consistent sequences. Next, we aligned these sequences to reference genes and used StringTie (version 2.1.4) to eliminate redundancy from the alignment. Specifically, StringTie merged only those alignments with differential 5’ exons to generate a non-redundant transcript set. Finally, we used GffCompare (version 0.12.1) to compare the non-redundant transcripts with known genome transcripts, enabling the discovery of novel transcripts and genes for further in-depth analysis.

### Differential gene and transcript analysis

To ensure comparability of transcript and gene expression levels across different transcripts, genes, and experimental groups, we used Transcripts Per Kilobase Million (TPM) as our standard metric. For each transcript and gene, we calculated the reads per kilobase (RPK) value by normalizing the read count by the length of the transcript or gene in kilobases. These RPK values were then scaled to TPM by dividing by one million, ensuring that the sum of all TPM values across samples was standardized. For quantitative assessment of transcript and gene expression, we utilized Salmon software (version 1.4.0). Following this, we performed differential expression analysis based on the expression quantifications derived from read count data for each sample. We employed DESeq2 (version 1.26.0) for this purpose, applying stringent criteria with an adjusted p-value threshold of less than 0.05 and an absolute log2 fold change greater than 1. If these criteria yielded an insufficient number of significantly differentially expressed transcripts or genes, we relaxed the threshold to a p-value of less than 0.05 while maintaining the same fold change criterion. This strategy allowed us to identify a comprehensive set of differentially expressed transcripts or genes for subsequent in-depth analysis.

### Functional enrichment analysis

Functional Enrichment Analysis utilizes statistical methods to determine if differentially expressed genes or transcripts are disproportionately represented within specific functional categories. This is accomplished through Gene Ontology (GO) and Kyoto Encyclopedia of Genes and Genomes (KEGG) Pathway enrichment analysis using hypergeometric tests^16,17^. These tests compare the observed distribution of differentially expressed genes across functional categories to a background distribution, typically comprising all annotated genes or transcripts. For this analysis, we employed the clusterProfiler software (version 3.14.3). The corrected p-value following multiple hypothesis testing, denoted as q value, ranges from 0 to 1. Values closer to zero indicate more statistically significant enrichment.

### AS analysis

The pre-mRNA generated through gene transcription undergoes various splicing patterns to yield distinct mature mRNAs, which are then translated into different proteins, contributing to the diversity of biological traits. This process, termed AS, is a crucial aspect of post-transcriptional mRNA processing. Utilizing suppa2 software, we identified seven primary types of AS events in the two sample groups: Skipping exon, Mutually exclusive exons, Alternative 5’ splice-site, Alternative 3’ splice-site, Retained intron, Alternative first exon, and Alternative last exon. Furthermore, we employed suppa2’s DiffSplice function to quantify the isoforms resulting from AS and pinpoint differential AS events between the groups. To investigate the potential roles of these differentially spliced events in skin and hair pigment production, transport, and distribution processes, we conducted GO functional and KEGG pathway enrichment analyses.

### Quantitative real-time PCR validation

From the same batch of skin samples that were sequenced, we retained total RNA for cDNA synthesis. To validate the sequencing data, we randomly selected 10 genes with significant differential expression: 5 upregulated genes and 5 downregulated genes, and subjected them to q-RT PCR analysis. Additionally, we chose 5 significant AS events detected in the sequencing data for validation via RT-PCR and agarose gel electrophoresis, to confirm the accuracy of the identified AS events. We designed specific primers for these genes using Primer Premier 5.0 software, referencing mouse gene sequences from GeneBank (primer sequences are provided in Supplementary Table 1). For statistical analysis of relative gene expression, we employed the 2^−∆∆Ct^ method, and determined statistical significance using One-way ANOVA, with p-values less than 0.05 indicating significance, respectively.

### Ethics statement

All animal handling was carried out in strict compliance with the established good practice guidelines of the relevant national and local animal welfare authorities. The experimental procedures were approved by the Ethics Committee of the Affiliated Changsha Central Hospital, Hengyang Medical School, University of South China, and were conducted in accordance with the principles of animal welfare and ethics to ensure the humane treatment of the animals involved in the research.

## Results

### **Histological analysis the skin of*Mitf*p.R324del knock-in mice**

**Fig. 1:**
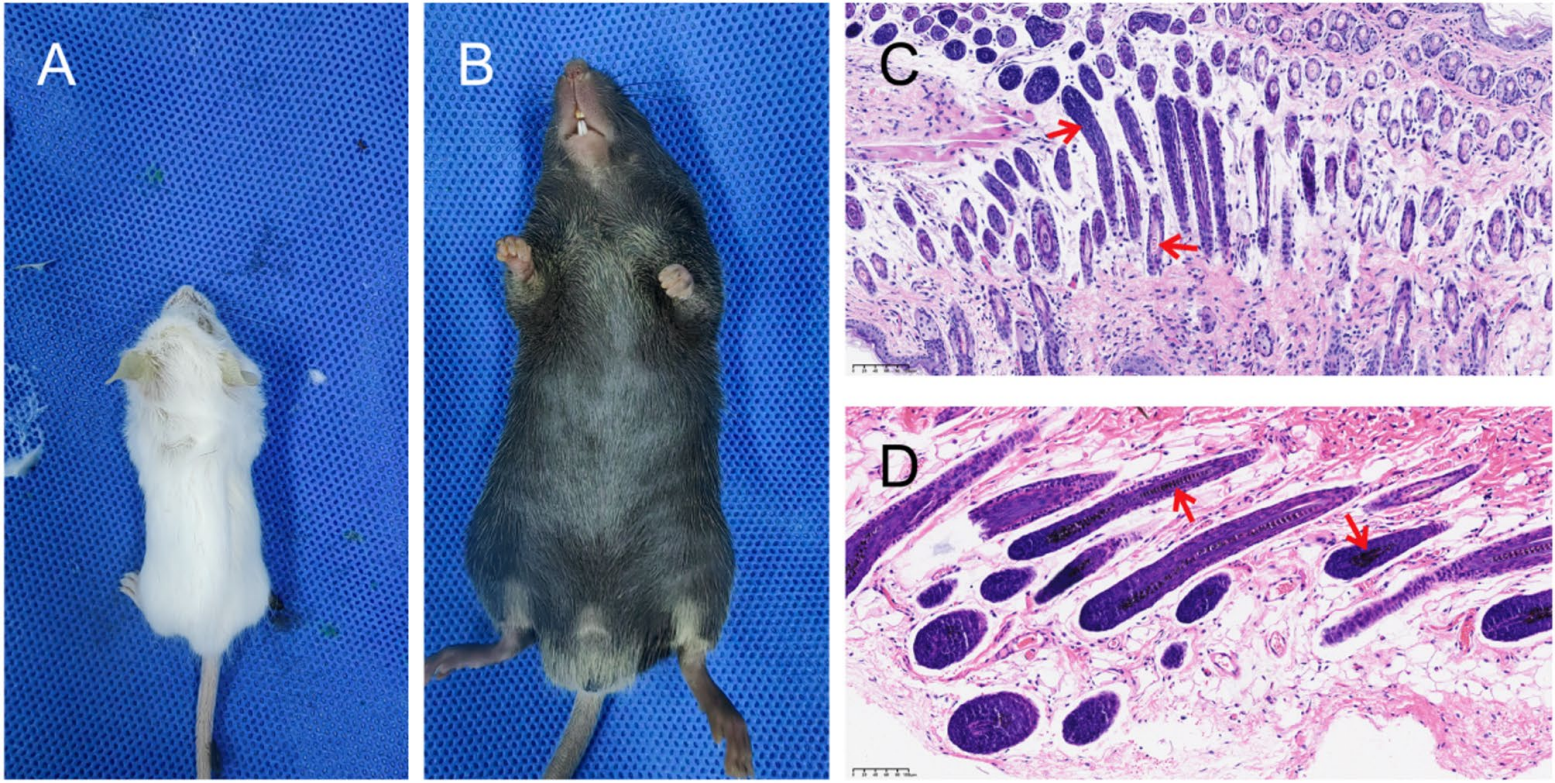
Phenotypes and H&E staining results of skin tissues in homozygous and wild-type mice with *Mitf* p.R324del mutation. (**A**) The homozygous *Mitf* p.R324del mutant mice exhibit a distinct appearance, characterized by pure white fur and a severe case of microphthalmia. (**B**) In contrast, wild-type mice feature pure black fur. (**C**) H&E staining of skin tissue from homozygous mice (magnification: 20X), a notable absence of melanin granules is observed in hair follicles and hair shafts (indicated by red arrows). (**D**) Conversely, H&E staining of skin tissue from wild-type mice reveals an abundance of melanin granules present in hair follicles and hair shafts (indicated by red arrows).

In the *Mitf* p.R324del knock-in mouse model, the homozygous mice exhibited a pure white coat, in contrast to the black coat of the wild-type mice (Fig. 1A-B). Then, we conducted histological analysis and H&E staining on paraffin sections of abdominal skin from various individuals. Our findings revealed that melanin granules were absent in the hair follicles and hair shafts of the skin of the pure white-coated mice (Fig. 1C-D). To further explore the potential causes of these phenotypic differences, we performed full-length transcriptome sequencing on abdominal skin samples from wild-type and homozygous mice, aiming to gain insights into the underlying mechanisms.

### Analysis of full-length transcriptome from ONT sequencing

To investigate the mechanisms underlying the anomalies in skin and hair pigment distribution induced by WS, and to discern the causative factors among genetically similar mice, we conducted full-length transcriptome sequencing on the abdominal skin of homozygous white-coated mice and wild-type black-coated mice using the ONT sequencing platform. Initially, we extracted and thoroughly assessed the sequencing data, performing quality control to determine basic metrics such as read number, N50, and average read length. Each sample underwent a rigorous quality check. To ensure analysis reliability, we filtered out low-quality and adapter sequences from the raw data, resulting in clean reads, as detailed in Table 1. Subsequently, isolate full-length sequences within the valid data (Supplementary Table 2). These sequences were then aligned to the reference genome (ENSEMBL 109) (Table 2). Further data refinement was achieved for clustering, deduplication, and correction, resulting in a consistent set of sequences (Supplementary Table 3). We compare non-redundant transcripts against known genome transcripts, which helped us identify novel transcripts and genes that enhance the existing annotation. Additionally, we performed a statistical analysis of all transcripts and genes, categorized as known and newly identified, to elucidate their distribution and frequency. This analysis resulted in density distribution plots of transcript distribution on the reference genome (Supplementary Fig. 1A-D).

**Table 1:** Statistics of sequencing data.

Sample	Type	Total base	Total reads	Max Len	Avg Len	N50	L50	N90	L90	meanQ
HM1	Raw	6,501,024,201	7,688,704	551,586	845.52	1159	1,510,323	374	5,412,940	11.14
HM1	Clean	6,085,738,369	7,204,085	206,957	844.76	1155	1,422,668	374	5,079,541	11.51
HM2	Raw	6,499,849,167	9,153,117	530,558	710.12	935	1,887,627	320	6,634,095	11.14
HM2	Clean	6,135,142,363	8,649,113	93,901	709.33	933	1,790,026	320	6,275,902	11.44
HM3	Raw	6,262,782,860	7,566,787	776,285	827.66	1147	1,471,037	364	5,317,715	10.59
HM3	Clean	5,819,111,570	7,012,659	89,513	829.8	1149	1,367,919	365	4,930,351	10.96
WT1	Raw	6,499,348,386	6,013,604	262,341	1,080.77	1429	1,294,857	561	4,233,808	10.6
WT1	Clean	6,017,811,405	5,508,532	31,625	1,092.45	1441	1,190,875	568	3,886,300	11.03
WT2	Raw	6,499,766,910	5,843,364	247,087	1,112.33	1370	1,361,834	602	4,282,738	11.34
WT2	Clean	6,158,589,773	5,502,246	28,613	1,119.28	1377	1,285,714	606	4,039,112	11.69
WT3	Raw	6,497,611,349	5,952,776	429,530	1,091.52	1331	1,416,148	590	4,393,955	10.57
WT3	Clean	5,877,359,827	5,324,230	107,498	1,103.88	1344	1,270,029	598	3,936,543	11.13

**Table 2:** Statistics of sequencing data aligned to the reference genome.

Sample	Total reads	Mapped reads	Map rate
HM1	5,477,027	4,713,601	86%
HM2	6,595,943	5,457,883	83%
HM3	5,187,832	4,386,179	85%
WT1	4,145,601	3,892,864	93.9%
WT2	4,429,402	4,273,135	96%
WT3	4,180,363	4,025,797	96.3%

### Differential expression genes and functional enrichment analysis

**Fig. 2:**
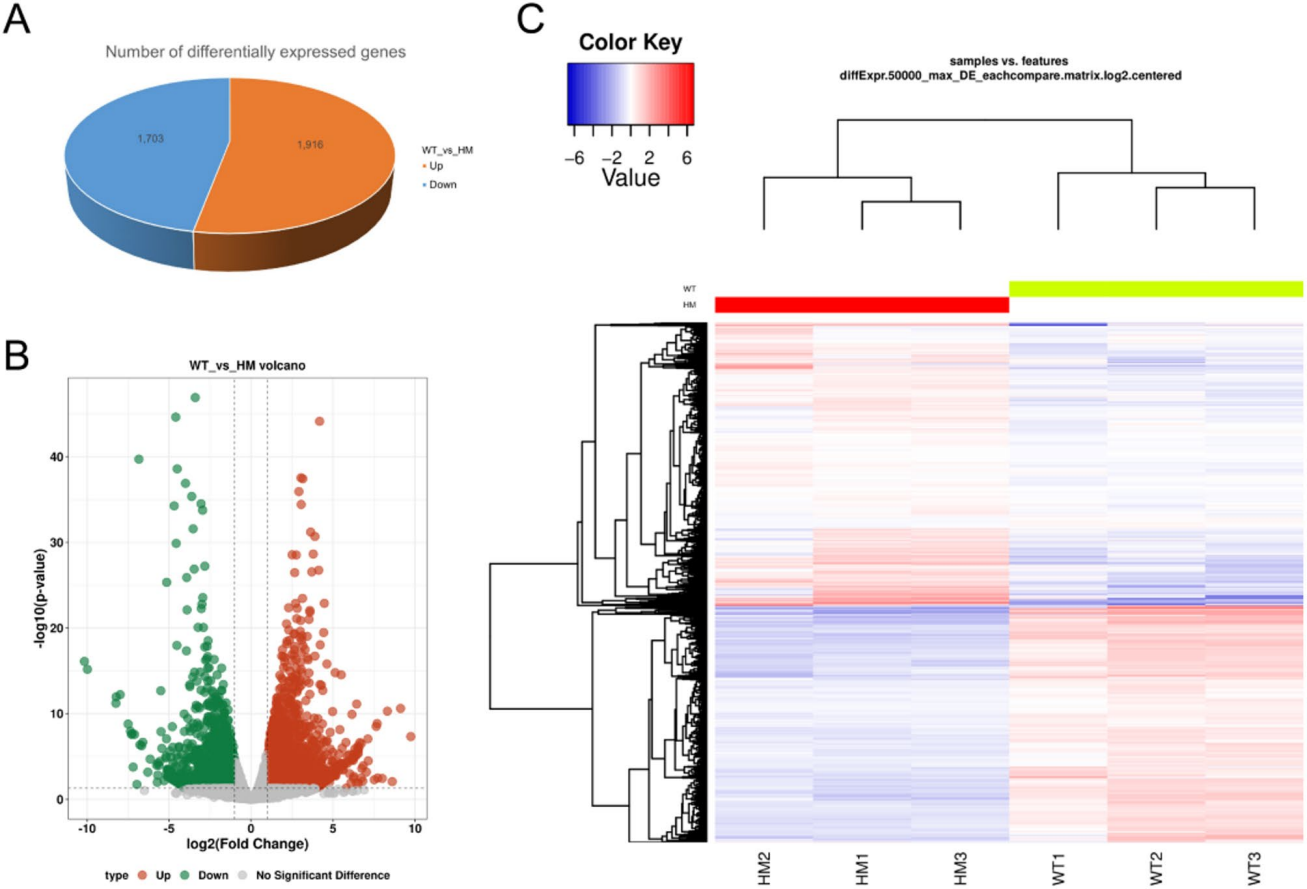
Inter-group analysis of differentially expression genes (WT VS HM). (**A**) Schematic representation of the quantity of differentially expression genes; (**B**) Volcano plot illustrating differentially expression genes; (**C**) Cluster analysis visualization of differentially expression genes.

To decipher the gene profiles and their interrelations within each sample, we initiated our study with a comprehensive analysis of gene expression, distribution, sample correlation, and Principal Component Analysis (Supplementary Fig. 2A-D). Building on the quantified gene expression data, we utilized DESeq2 software (Version 1.26.0) to perform differential gene expression analysis between the study groups. We applied a threshold of an adjusted p-value (padj) less than 0.05 and an absolute log2 fold change greater than 1, revealing 3619 genes with differential expression. Specifically, when contrasting the homozygous (HM) group with the wild-type (WT) group, we identified 1916 upregulated and 1703 downregulated genes (Fig. 2A-C). The most significantly upregulated genes were *Myh7*, *Calca*, *Fabp2*, *Tat*, *Apoa4*, *Aldob*, *Igfn1*, *Tnni1*, *Tnnc1*, and *Myl3*. Conversely, the most downregulated genes were *Tpsb2*, *Mlana*, *Lep*, *A4gnt*, *Mylk4*, *Mup3*, *Cartpt*, *Cypzg1*, *Amigo3*, and *Klra1* (Supplementary Table 4). Furthermore, a subset of genes implicated in melanin synthesis and transport, and Mitf-associated pathways, notably *Mlana*, *Dct*, *Slug*, Trp53, Wnt4, Fzd2, Adh1, Aldh3a1, Fgf7, Flt3, and Mcoln3, exhibited significant downregulation in the HM group (Table 3). Interestingly, numerous genes intimately linked to *Mitf* function and melanin production, including *Lef1*, *Arcn1*, *Edn3*, *Pax3*, *Tyrp1*, *Pmel*, *Lyst*, *Rab38*, *Myo5a*, *Myo7a*, *Rab27a*, *Ostm1*, *Tyr*, *Kit*, *Silv*, *Bcl2*, and *Sox10*, did not exhibit notable expression changes. These findings provide a substantial framework for further investigating the mechanisms underlying skin and fur pigmentation anomalies in *Mitf* p.R324del knock-in mice.

**Table 3:** Statistics of differentially expressed pigment-related genes.

GeneID	Treatment	Control	baseMean Treatment	baseMean Control	BaseMean	log2FoldChange	lfcSE	stat	pvalue	padj	diffType	gene_ name
ENSMUSG00000024806	HM	WT	0	25.98213442	12.991067	− 7.192544049	1.91429	− 3.75728	0.00017	0.00208	Down	Mlana
ENSMUSG00000022129	HM	WT	2.57104691	30.8458305	16.708439	− 3.586392793	0.70344	− 5.09834	3.43E− 07	1.08E− 05	Down	Dct
ENSMUSG00000022676	HM	WT	31.17406978	76.57225161	53.873161	− 1.298070954	0.34108	− 3.8058	0.00014	0.00177	Down	Snai2
ENSMUSG00000059552	HM	WT	6.010444813	18.51223697	12.261341	− 1.612559209	0.64758	− 2.49013	0.01277	0.0608	Down	Trp53
ENSMUSG00000036856	HM	WT	15.51951647	31.58380871	23.551663	− 1.021626391	0.47684	− 2.14248	0.03215	0.11826	Down	Wnt4
ENSMUSG00000050288	HM	WT	14.80529625	34.22828455	24.51679	− 1.199981121	0.49045	− 2.44667	0.01442	0.06637	Down	Fzd2
ENSMUSG00000074207	HM	WT	73.27286115	152.5149987	112.89393	− 1.062867468	0.34532	− 3.07791	0.00208	0.01525	Down	Adh1
ENSMUSG00000019102	HM	WT	32.00379855	183.7198996	107.86185	− 2.531561367	0.37416	− 6.76596	1.32E−11	1.21E−09	Down	Aldh3a1
ENSMUSG00000027208	HM	WT	20.1571916	49.52701292	34.842102	− 1.294509365	0.40977	− 3.15908	0.00158	0.01232	Down	Fgf7
ENSMUSG00000042817	HM	WT	4.196876978	21.07098111	12.633929	− 2.336205591	0.73734	− 3.16844	0.00153	0.01199	Down	Flt3
ENSMUSG00000036853	HM	WT	0	3.343228201	1.6716141	− 4.236617259	1.81871	− 2.32946	0.01983	1	Down	Mcoln3

**Fig. 3:**
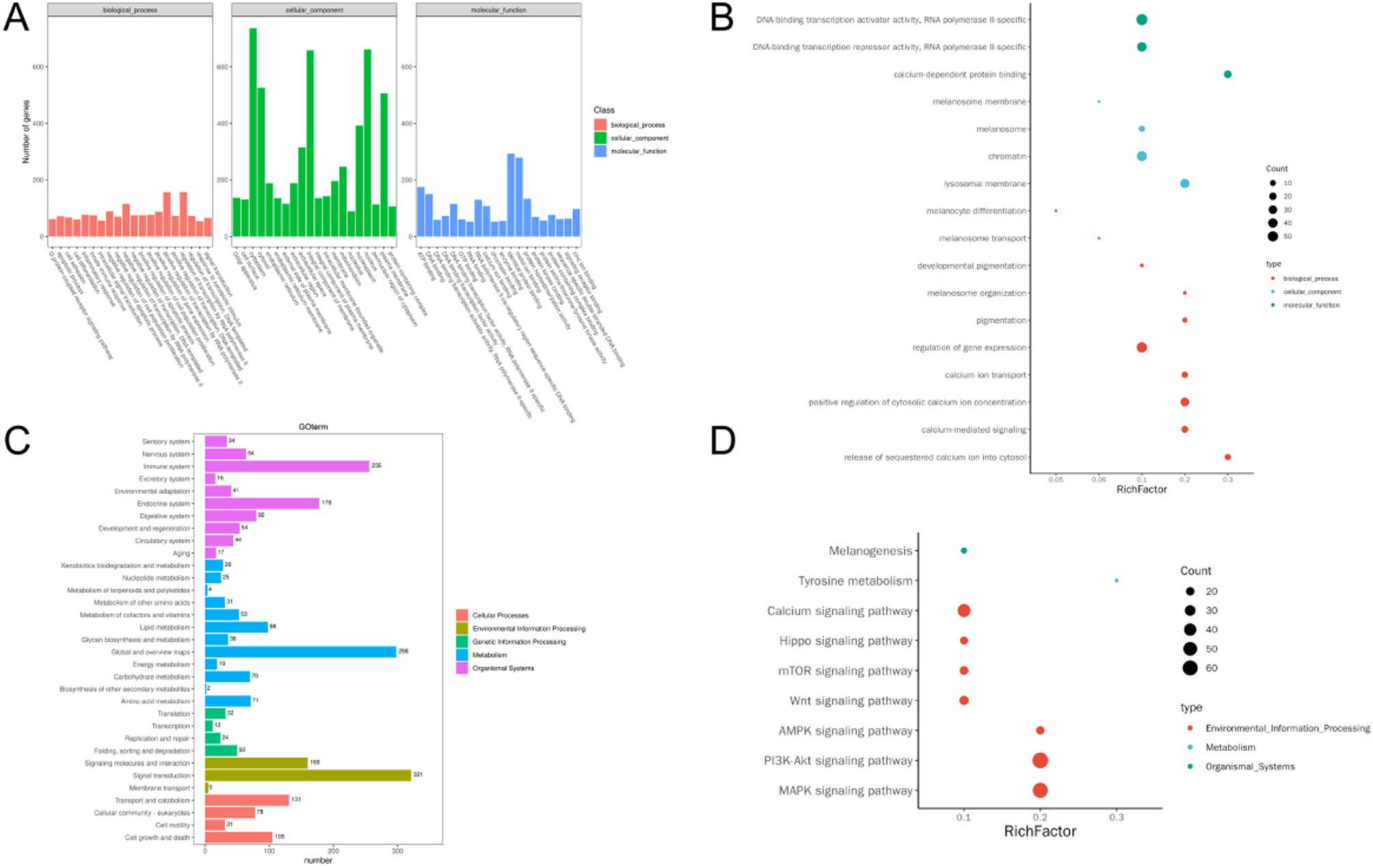
Results of functional enrichment analysis for GO terms and KEGG pathways in differentially expression genes. (**A**) Comprehensive GO functional enrichment analysis outcomes for differentially expression genes; (**B**) GO functional enrichment analysis outcomes specifically for differentially expression genes linked to *Mitf* and pigmentation; (**C**) Comprehensive KEGG pathway enrichment analysis outcomes for differentially expression genes; (**D**) KEGG pathway enrichment analysis outcomes specifically for differentially expression genes linked to *Mitf* and pigmentation.

To further understand the biological functions of the differentially expressed genes, we conducted GO Term and KEGG pathway enrichment analysis. GO term enrichment analysis showed that the differentially expressed genes were mainly enriched in processes such as cytoplasm, nucleus, integral component of membrane, identical protein binding, metal ion binding, and positive regulation of transcription by RNA polymerase II (Fig. 3A). Further analysis of *Mitf* and pigment-related GO terms revealed enrichment in multiple pigment-related GO terms, including pigmentation, melanocyte differentiation, melanosome, developmental pigmentation, chromatin, DNA-binding transcription repressor activity, RNA polymerase II-specific, and others (Fig. 3B). KEGG pathway enrichment analysis showed that the differentially expressed genes were enriched in signaling pathways such as Signal transduction, Global and overview maps, Immune system, Endocrine system, Signaling molecules and interaction, and others (Fig. 3C). Similarly, in KEGG pathway enrichment analysis, significantly different genes were found to be enriched in pathways closely related to pigments and pigment cells, such as Melanogenesis, Tyrosine metabolism, MAPK signaling pathway, and Wnt signaling pathway (Fig. 3D). These results indicate that *Mitf* p.R324del mice may have some obstacles in melanocyte-related functions and pigment synthesis, leading to abnormal pigment distribution in their fur.

### Differentially expression transcripts and AS events analysis

**Fig. 4:**
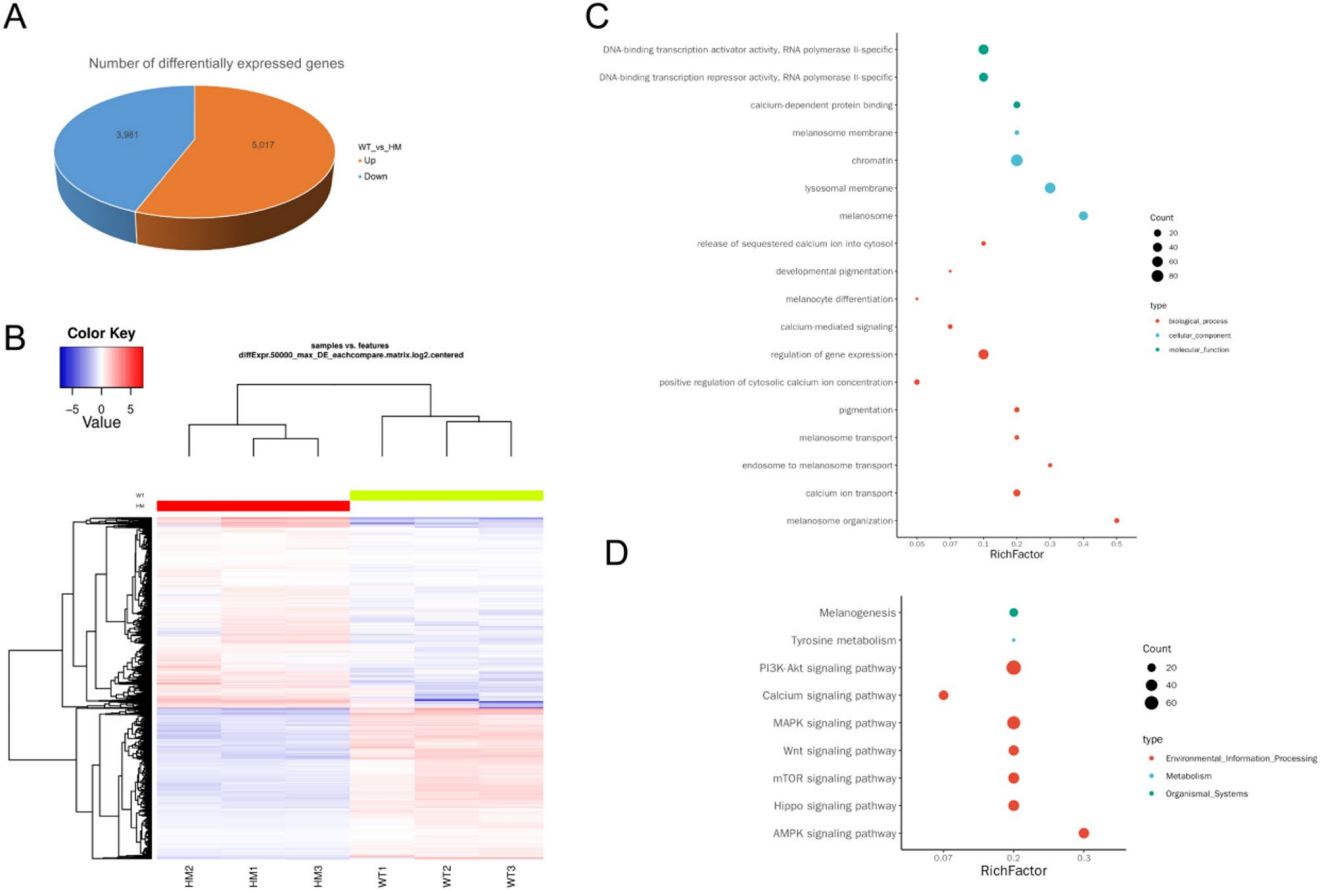
Results of inter-group analysis for differentially expression transcripts and alternative splicing (AS) events: (**A**) Schematic illustration of the quantity of differentially expressed transcripts; (**B**) Cluster analysis visualization of differentially expressed transcripts; (**C**) Schematic representation of GO terms enrichment analysis for differential AS events associated with pigmentation-related genes; (**D**) Enrichment analysis outcomes of KEGG pathways for differential AS events in pigmentation-related genes.

Transcripts, representing mature mRNAs synthesized from a single gene, possess the ability to encode a single or multiple proteins. Due to the intricacies of splicing mechanisms, a gene can generate multiple transcript isoforms, leading to distinct expression patterns across various tissues or cell types. To delve deeper into these complexities, we utilized full-length transcriptome sequencing to scrutinize transcript expression discrepancies between two sample groups. Our exhaustive analysis uncovered 8998 differentially expressed transcripts. When comparing the HM group to the WT group, we observed 5017 transcripts with heightened expression and 3981 with diminished expression (Fig. 4A). Additionally, we conducted a clustering analysis to discern patterns among the differentially expressed transcripts and the samples (Fig. 4B).

AS is a pivotal post-transcriptional process that enables a single pre-mRNA transcript to be processed into numerous mature mRNA isoforms, potentially leading to distinct protein variants. This process plays a crucial role in biological diversity. Using the suppa2 software, we identified seven distinct AS events: Alternative 3’ splice site (A3), Alternative 5’ splice site (A5), Alternative First exon (AF), Alternative Last exon (AL), Mutually exclusive exon (MX), Retained intron (RI), and Skipping exon (SE). In our comparative analysis between the two sample groups, we discovered a statistically significant number of differentially spliced events across each category (*P* < 0.05): A3 with 97 events, A5 with 85, AF with 101, AL with 42, MX with 8, RI with 44, and SE with 184 (Table 4).

**Table 4:** Numbers of significant AS events between wild-type and homozygous mice.

AS_type	Diff_AS_num	Significant_Diff_AS_num
Alternative 3′ splice site (A3)	1379	97
Alternative 5′ splice site (A5)	1264	85
Alternative first exon (AF)	2146	101
Alternative last exon (AL)	408	42
Mutually exclusive exon (MX)	107	8
Retained intron (RI)	766	44
Skipping exon (SE)	2751	184

To investigate the potential role of AS in skin and hair pigmentation, we analyzed the occurrence of AS events in differentially expressed pigment-related genes between the two groups. Unfortunately, we did not uncover significant differential AS events in the relevant genes. However, functional enrichment analysis further indicated that these significant AS events were associated with various Gene Ontology (GO) terms pertinent to pigmentation pathways, such as pigmentation, developmental pigmentation, melanosome activity, and transport mechanisms (Fig. 4C). Furthermore, KEGG pathway analysis linked the differentially spliced events to crucial pathways like Melanogenesis, Tyrosine metabolism, and the MAPK and Wnt signaling pathways (Fig. 4D). These findings suggest that AS may influence melanocyte generation, synthesis, and transport, thereby affecting pigment distribution in the skin and hair of *Mitf* p.R324del mice, potentially leading to the observed pigmentation anomalies.

### qRT-PCR validation sequencing results

**Fig. 5:**
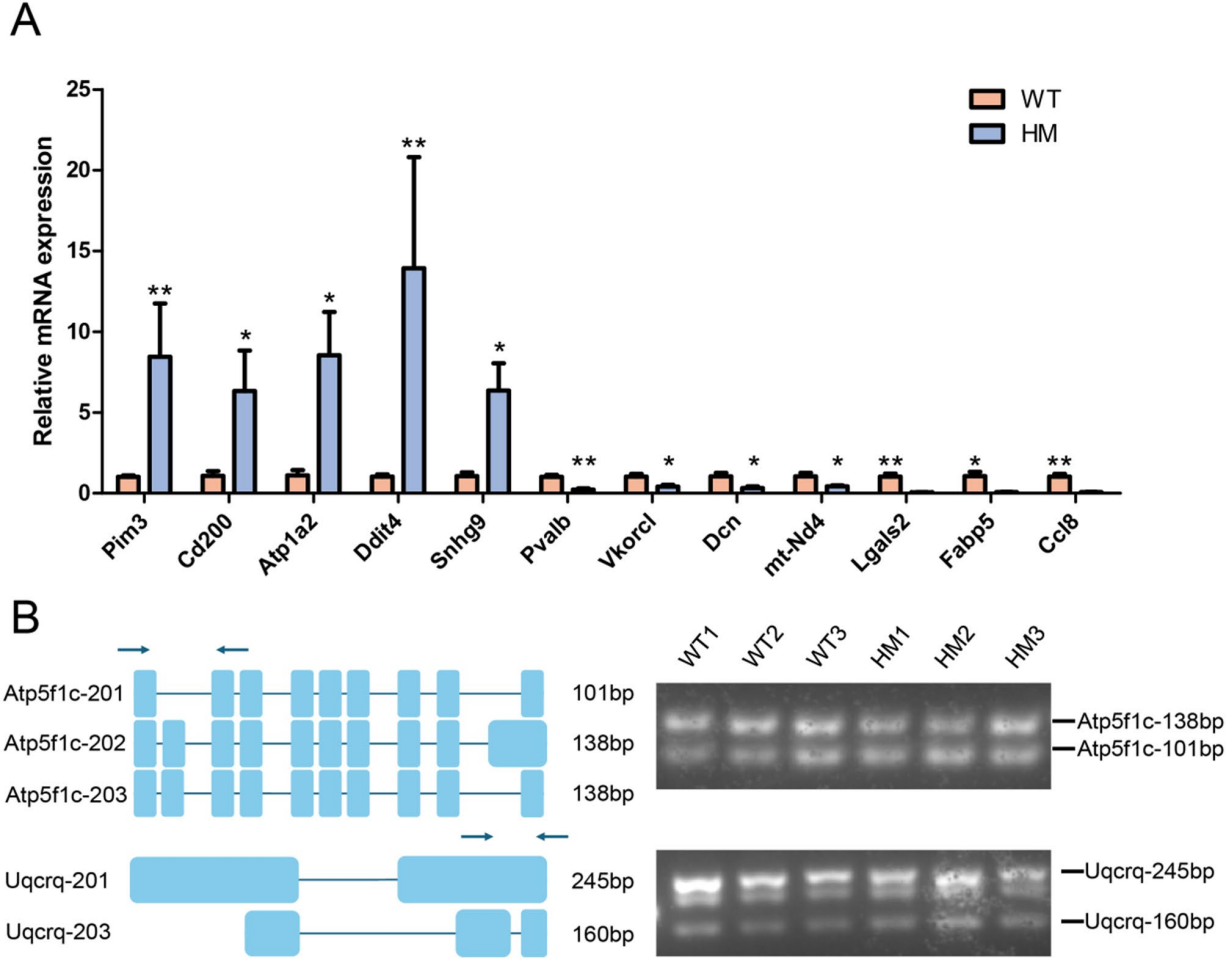
qRT-PCR validation outcomes of sequencing data. (**A**) Confirmation results of differentially expression transcripts (with significance levels of * *P* < 0.05 and ** *P* < 0.01). (**B**) Agarose gel electrophoresis results for differential AS events, Arrows indicate the positions of primers on selected genes.

To verify the precision of our sequencing outcomes, we randomly chose 5 genes that were significantly upregulated and another 5 that were significantly downregulated for qRT-PCR validation, as illustrated in Fig. 5A. Additionally, we randomly picked 5 notable differential AS events for RT-PCR and agarose gel electrophoresis validation, as depicted in Fig. 5B. Through agarose gel electrophoresis analysis of PCR products, we confirmed that the *Atp5f1c* and *Uqcrq* genes underwent distinct patterns of alternative splicing events between the two sample groups, generating different transcript variants: *Atp5f1c-201*,* Atp5f1c-202*,* Atp5f1c-203 and Uqcrq-201*,* Uqcrq-203*. These findings were consistent with the results obtained from sequencing analysis. The results obtained from these validations were in agreement with our sequencing data, thereby affirming the credibility of our sequencing results.

## Discussion

Waardenburg syndrome (WS) represents a paradigmatic example of autosomal dominant genetic disorders, with certain mutations exhibiting recessive inheritance patterns^18^. WS is divided into four distinct types, with approximately 15% of WS2 cases attributed to mutations in the *MITF* gene^19^. The widely accepted “neural crest development deficiency theory” provides a robust framework for understanding WS pathogenesis, positing that mutations disrupt neural crest cell function, melanocyte development, and terminal differentiation, ultimately leading to the characteristic pigmentary abnormalities observed in WS^20^. The *Mitf* gene, a member of the Myc supergene family, encodes a transcription factor characterized by a highly conserved basic helix-loop-helix leucine zipper (bHLH-Zip) structure. This structural motif enables *Mitf* to generate multiple isoforms through AS, which are differentially expressed across various cell types^21^. *Mitf* plays a pivotal role in melanocyte development, differentiation, survival, and melanin synthesis and transport, making it a central player in pigmentation biology^22^.

Mice have emerged as a primary model for genetic studies due to their accessibility, rapid breeding cycles, low experimental costs, and suitability for extensive and repetitive testing. The skin and hair color of mice are influenced by both genetic and environmental factors, rendering them an invaluable tool for investigating genes related to pigmentation^23,24^.In this study, we employed CRISPR/Cas9 gene editing to generate a precise *Mitf* p.R324del knock-in mouse model, mimicking the human *MITF* p.R217del mutation associated with WS2A. Homozygous mutant mice exhibited a striking white coat color, providing a robust model to investigate the molecular mechanisms underlying pigmentary abnormalities. To elucidate these mechanisms, we conducted full-length transcriptome sequencing on abdominal skin samples from homozygous and wild-type mice.

The color of mammalian skin and hair is primarily determined by the relative proportions of two melanin types produced by melanocytes: eumelanin and pheomelanin^25,26^. Currently, over a hundred pigment-related genes have been identified and categorized into six functional groups, including melanocyte development and differentiation, melanosomal components, organelle biogenesis, organelle transport, control of pigment-type switching, and systemic effects^27^. Notably, most of these genes are expressed in mouse skin, making it an ideal system for studying pigmentation^28^. Our study revealed differential expression of numerous pigment-related genes in the skin of homozygous versus wild-type mice. Including *Mlana*, *Dct*, *Slug*, *Trp53*, *Wnt4*, *Fzd2*, *Adh1*, *Aldh3a1*, *Fgf7*, *Flt3*, and *Mcoln3*, among others. These genes are likely contributors to the observed differences in pigment deposition, with their coordinated regulation playing a critical role in melanocyte development and function^29,30^.

Among these, *Dct* (dopachrome tautomerase) is a key enzyme in melanin biosynthesis in melanin biosynthesis, catalyzing the isomerization of dopachrome to DHICA (5,6-dihydroxy-indole-2-carboxylic acid). As one of the three essential enzymes in melanin biosynthesis, *Dct* is directly regulated by *Mitf* and influences both the quantity and quality of melanin^31^. Similarly, *Mlana* (melan-A), which is specifically expressed in melanocytes, melanomas, and retinal pigment epithelial cells, plays a pivotal role in melanocyte differentiation under the guidance of the *Mitf* gene^32–34^. *Mlana* is one of the key structural proteins for mature melanosome structure^35^. In our study, *Mlana* expression was significantly down-regulated in the skin of homozygous mice, suggesting impaired melanocyte differentiation and melanosome maturation. Of course, further investigation is necessary to elucidate the precise roles of these genes in melanocyte development and pigment deposition, and their contributions to the observed phenotypic differences. Additionally, *Snai2* (slug), a key gene implicated in neural crest cell development and pigmentation, was markedly down-regulated in our model. *Snai2* is known to be transcriptionally regulated by *Mitf*, further underscoring the central role of *Mitf* in melanocyte biology^36–38^.

We hypothesize that the *Mitf* p.R324del mutation disrupts critical functions of the *Mitf* gene, particularly those related to melanocyte migration, differentiation, and melanin synthesis. These disruptions, once established, may be irreversible, leading to the observed phenotype of pure white fur in homozygous mice. These insights pave the way for further studies to understand the intricacies of melanocyte genetics and their influence on pigment-related disorders. Based on our results, we believe that the *Mitf* p.R324del mutation leads to the downregulation of downstream *Snai2*, *Mlana*, and *Dct* gene expression, affecting melanocyte differentiation, melanosome structure, and melanin biosynthesis, ultimately resulting in the pigmentary abnormalities observed in our model.

Functional enrichment analysis of differentially expressed genes (DEGs) provided profound insights into their roles in pigmentation. GO and KEGG pathway analyses revealed significant enrichment in processes such as pigmentation, melanocyte differentiation, melanosome, developmental pigmentation, chromatin, DNA-binding transcription repressor activity specific to RNA polymerase II, and key pathways included Melanogenesis, Tyrosine metabolism, MAPK signaling pathway, and Wnt signaling pathway, all of which are critical for melanocyte function and pigmentation^39^. These findings highlight the complex regulatory networks governing pigmentation and suggest that disruptions in these pathways contribute to the diverse pigment phenotypes observed in WS.

AS is a fundamental process in gene regulation, affecting over 90% of human genes and generating a diverse array of mRNA isoforms with distinct functions^40^. Leveraging Oxford Nanopore Technologies’ full-length transcriptome sequencing, we identified and analyzed AS events in the skin of *Mitf* p.R324del mice. While no significant differences in AS events were observed in pigment-related genes, functional enrichment analysis revealed that AS events in other genes were associated with pigmentation-related processes, including developmental pigmentation, melanosome activity, transport from endosome to melanosome, melanocyte differentiation, and Melanogenesis, Tyrosine metabolism, MAPK signaling, Wnt signaling pathways. These findings suggest that AS events may contribute to the regulation of pigmentation, albeit indirectly, by modulating the expression of genes involved in melanocyte function.

Although this study uncovered several intriguing findings and yielded preliminary results, certain limitations remain that warrant further investigation, functional validation, and in-depth analysis. For instance, a major limitation is the lack of direct evidence supporting functionally relevant alternative splicing (AS) events in pigmentation-related genes. Additionally, due to sample constraints and the scope of this study, we were unable to establish a direct link between the observed AS events and the phenotypic manifestations in this model. Furthermore, although we detected significant downregulation of key *Mitf* downstream target genes (e.g., *Dct*, *Mlana*, and *Snai2*), their interactive mechanisms and functional roles in this model remain unexplored. Moreover, the potential regulatory effects of non-coding RNAs (ncRNAs) were not investigated in this study. These unresolved questions will serve as key focuses for future research.

In summary, our study provides novel insights into the molecular mechanisms underlying pigmentary abnormalities in WS mice caused by *Mitf* p.R324del mutation. The significant down-regulation of *Dct*, *Mlana*, and *Snai2* highlights the critical role of *Mitf* in the differentiation of neural crest cells into melanocytes, the structure of melanosomes, and the biosynthesis of melanin. Furthermore, our findings suggest that AS events may also contribute to the regulation of pigmentation, adding another layer of complexity to the genetic networks governing melanocyte biology. These results not only enhance our understanding of WS pathogenesis but also provide a foundation for future research with Mitf-related pathways.

## Supplementary Information

Below is the link to the electronic supplementary material.

Supplementary Material 1

Supplementary Material 2

## Data Availability

Sequence data that support the findings of this study have been deposited in the SRA https://www.ncbi.nlm.nih.gov/sra/PRJNA1244119. For further information contact the corresponding author.
