# Supplementary material for: Full length transcriptomic profiling reveals insights into the white coat phenotype in Waardenburg syndrome mice harboring the Mitf R324del mutation
Source: Sci Rep. 2025 Jul 31;15:28012. doi: 10.1038/s41598-025-13359-8 (PMC12314077; doi:10.1038/s41598-025-13359-8)
Supplement: Supplementary file 1 — Supplementary Material 1 [file 41598_2025_13359_MOESM1_ESM.docx]

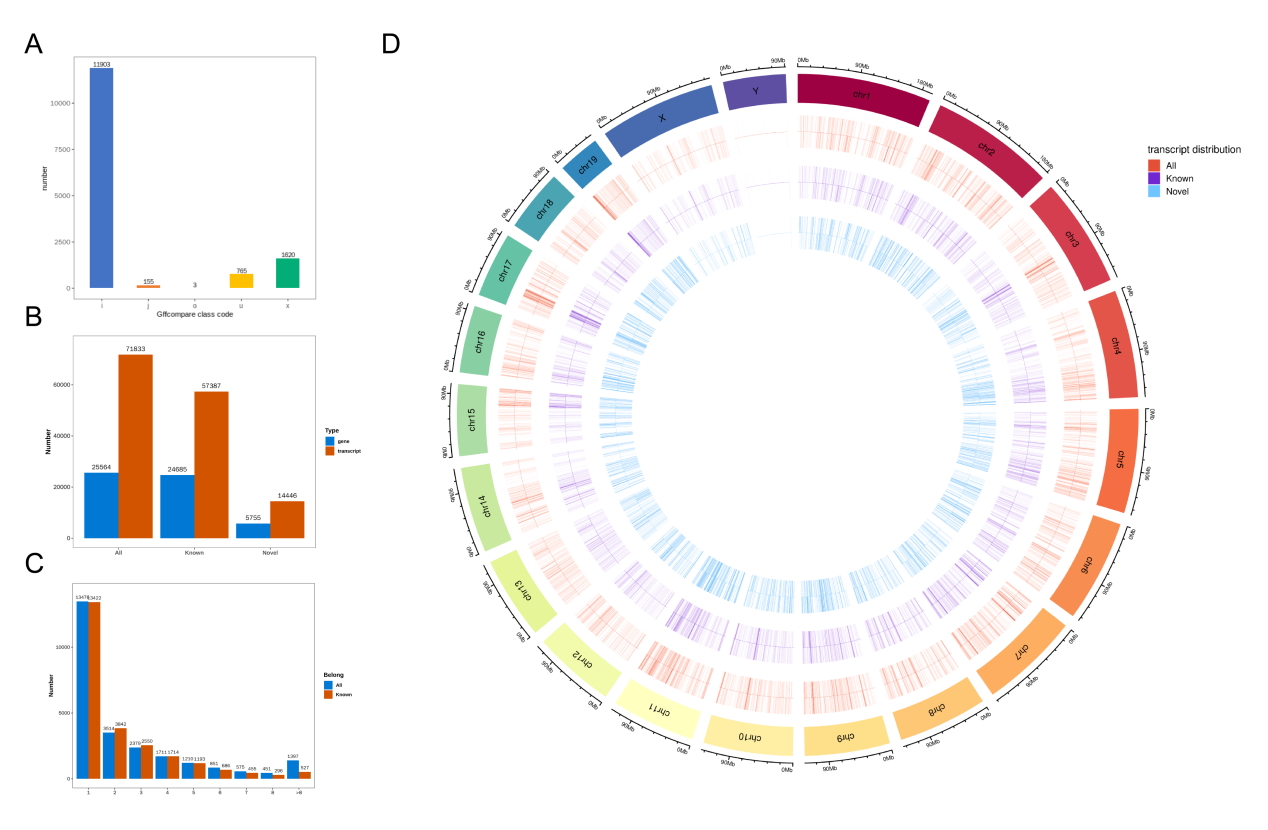


Supplementary Figure 1: Results of new genes and new transcripts in samples.

A. Distribution diagram of new transcript varieties in Samples ('o' represents overlapping segments with other reference exons on the same strand, 'j' indicates the existence of at least one matched multi-exon, 'x' signifies exon overlap on the antisense strand, 'i' denotes being entirely enclosed within the intron of a reference transcript, and 'u' stands for unidentified new transcripts).

B. Number chart illustrating genes and transcripts count in Samples (Known: previously identified, Novel: newly discovered, and All-inclusive of both known and novel).

C. Number chart showing genes with varying quantities of transcripts in samples (Known :previously identified, All: inclusive of both known and novel).

D. Density distribution diagram of transcripts across the reference genome in samples (Arranged from outermost to innermost, it displays chromosomes, all transcripts, known transcripts, and new transcripts, while also differentiating between the positive and negative strands).


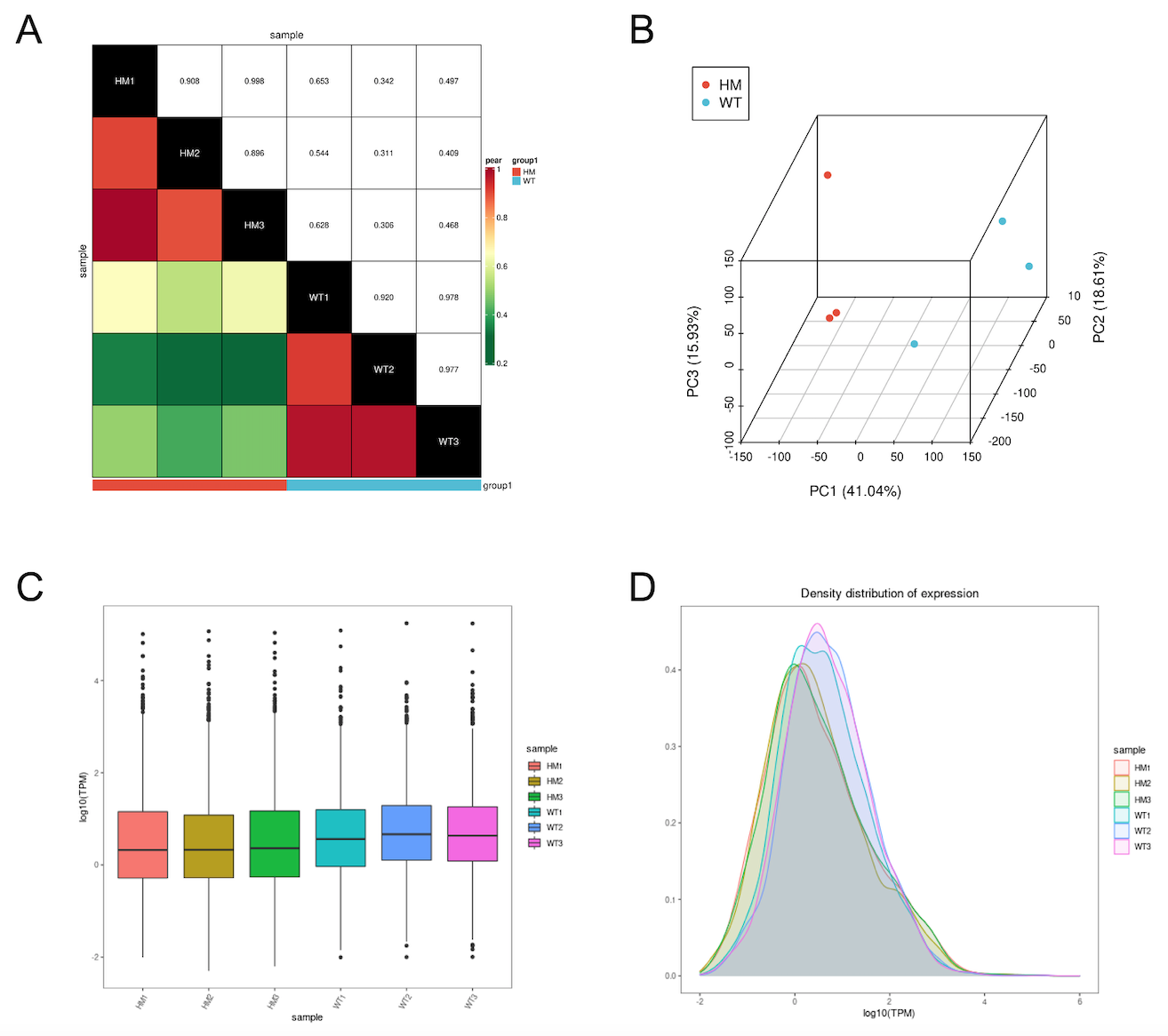


Supplementary Figure 2：Comprehensive analysis of sequencing data across various samples

A. A Pearson correlation coefficient heatmap illustrating the relationships between different samples.
B. A schematic diagram depicting the principal component analysis for each sample.
C. A TPM box plot showcasing the distribution of TPM values across different samples.
D. A TPM density distribution diagram illustrating the density of TPM values for different samples (HM: homozygous WT: wild type).

| Table S1. Primers sequence for gene identification | |
| --- | --- |
| gene name | sequence |
| Pim3 -Forward | CCAGAGTGCCAGCAGCTTAT |
| -Reverse | GCTCCGTCGTCAGTATCCAG |
| Cd200 -Forward | CCATCGTGGAAGAGCTCAGAC |
| -Reverse | CGGTAACCAGGGAACACTCCA |
| Atp1a2 -Forward | CGGGAGCCATAAGGGTTTGT |
| -Reverse | GCACTGACTTGGCTGTTGTG |
| Ddit4 -Forward | TGCCCACCTTTCAGTTGACC |
| -Reverse | GACTGGCTGTAACCAGGGAC |
| Snhg9 -Forward | CCAGCCGGAAGGACGTATAA |
| -Reverse | AGGACAATTCTTGCGGGAGG |
| Pvalb -Forward | CCAACAGGATCTCCCACCAG |
| -Reverse | GGCTCCTATCGCCTTCTTGA |
| Vkorcl -Forward | TGTACTGTCGACATGGGCAC |
| -Reverse | AGCGCGCGGTAATTTTCATC |
| Dcn -Forward | TCCACACCTGCAAACCCTTT |
| -Reverse | ATCCGGGTATTTGCCACAGG |
| mt-Nd4 -Forward | TGGCCTCACATCATCACTCC |
| -Reverse | CATTTGAAGTCCTCGGGCCA |
| Lgals2 -Forward | GCCCTTTCATGGGAGAGTGT |
| -Reverse | TCTGTGCTGGGCTCTCTTTG |
| Fabp5 -Forward | TGTGTGTGGGTTCTTCAAGG |
| -Reverse | GCAAGCTTAAATGACGGGGC |
| Ccl8 -Forward | CTACGCAGTGCTTCTTTGCC |
| -Reverse | GAAAGCAGCAGGTGACTGGA |
| Atp5f1c -Forward | CCTTGACTTTCAACCGCACC |
| -Reverse | CAGCCTGCGCATTCTTCTTC |
| Uqcrq -Forward | CGGAAGTGACGGGCGAAA |
| -Reverse | TGGGGATGCCTTTGCTGAAA |

| Table S2. Full-length sequences of each sample | | | | | | |
| --- | --- | --- | --- | --- | --- | --- |
| Sample Name | Seq Num | Mean Length(bp) | | N50(bp) | | Max Length(bp) |
| HM1 | 5,477,027 | 606.82 | | 968 | | 17,751 |
| HM2 | 6,595,943 | 498.02 | | 805 | | 13,081 |
| HM3 | 5,187,832 | 596.77 | | 976 | | 40,716 |
| WT1 | 4,145,601 | 860.98 | | 1,217 | | 14,875 |
| WT2 | 4,429,402 | 889.67 | | 1,171 | | 15,157 |
| WT3 | 4,180,363 | 874.44 | | 1,149 | | 13,674 |
| Table S3. Consistent sequence of each sample | | | | | |  |
| Sample Name | Seq Num | Mean Length(bp) | N50(bp) | | Max Length(bp) |  |
| HM1 | 22,866 | 636.12 | 908 | | 6,730 |  |
| HM2 | 24,691 | 519.53 | 732 | | 6,037 |  |
| HM3 | 21,257 | 648.36 | 943 | | 6,490 |  |
| WT1 | 17,334 | 846.22 | 1,069 | | 6,332 |  |
| WT2 | 17,811 | 963.92 | 1,256 | | 6,764 |  |
| WT3 | 16,591 | 935.63 | 1,205 | | 6,807 |  |

| Table S4. Statistics of the top ten most significantly up-regulated and down-regulated genes in sequencing results | | | | | | | | | | | | | |
| --- | --- | --- | --- | --- | --- | --- | --- | --- | --- | --- | --- | --- | --- |
| GeneID | treatment | control | baseMean  Treatment | baseMean  Control | baseMean | log2FoldChange | lfcSE | stat | pvalue | padj | diffType | | gene_name |
| ENSMUSG00000053093 | HM | WT | 159.5555179 | 0 | 79.77775894 | 9.747948107 | 1.783986022 | 5.464139286 | 4.65E-08 | 1.88E-06 | | Up | Myh7 |
| ENSMUSG00000030669 | HM | WT | 572.8481269 | 1.053808754 | 286.9509678 | 9.120237269 | 1.365998525 | 6.67660843 | 2.45E-11 | 2.08E-09 | | Up | Calca |
| ENSMUSG00000023057 | HM | WT | 141.4088437 | 0.354761406 | 70.88180256 | 8.61165162 | 3.270354318 | 2.633247282 | 0.008457275 | 0.044409843 | | Up | Fabp2 |
| ENSMUSG00000001670 | HM | WT | 59.00678211 | 0 | 29.50339106 | 8.31645348 | 1.265936286 | 6.569409196 | 5.05E-11 | 4.15E-09 | | Up | Tat |
| ENSMUSG00000032080 | HM | WT | 47.70321608 | 0 | 23.85160804 | 8.006151207 | 2.751446282 | 2.909797389 | 0.003616631 | 0.023333322 | | Up | Apoa4 |
| ENSMUSG00000028307 | HM | WT | 146.5429525 | 0.699047347 | 73.62099992 | 7.726052781 | 2.620177324 | 2.948675538 | 0.003191388 | 0.021123778 | | Up | Aldob |
| ENSMUSG00000051985 | HM | WT | 33.99308563 | 0 | 16.99654281 | 7.517547939 | 2.68723205 | 2.797506058 | 0.00514988 | 0.03078752 | | Up | Igfn1 |
| ENSMUSG00000026418 | HM | WT | 51.22684125 | 0.344285941 | 25.7855636 | 7.148090289 | 1.719654477 | 4.156701467 | 3.23E-05 | 0.000519324 | | Up | Tnni1 |
| ENSMUSG00000091898 | HM | WT | 193.0122799 | 1.40857016 | 97.21042503 | 7.12852317 | 1.332070766 | 5.35145981 | 8.72E-08 | 3.28E-06 | | Up | Tnnc1 |
| ENSMUSG00000059741 | HM | WT | 89.53711785 | 0.688571882 | 45.11284487 | 7.036148025 | 2.063721223 | 3.409446947 | 0.000650947 | 0.006155806 | | Up | Myl3 |
| ENSMUSG00000079853 | HM | WT | 0 | 5.923218257 | 2.961609128 | -5.065736477 | 1.610541842 | -3.145361608 | 0.001658817 | 1 | | Down | Klra1 |
| ENSMUSG00000032593 | HM | WT | 0.295051497 | 11.92244078 | 6.108746139 | -5.111539004 | 1.410729862 | -3.623329414 | 0.000290835 | 0.003210841 | | Down | Amigo3 |
| ENSMUSG00000049685 | HM | WT | 8.71043488 | 343.1313758 | 175.9209053 | -5.300127182 | 1.951070459 | -2.716522696 | 0.006597167 | 0.036969272 | | Down | Cyp2g1 |
| ENSMUSG00000021647 | HM | WT | 0.637354371 | 33.7244192 | 17.18088679 | -5.722435301 | 2.222708699 | -2.574532283 | 0.010037572 | 0.050566636 | | Down | Cartpt |
| ENSMUSG00000066154 | HM | WT | 0 | 9.655310271 | 4.827655135 | -5.767593401 | 1.482829988 | -3.889585081 | 0.000100416 | 0.001335414 | | Down | Mup3 |
| ENSMUSG00000044951 | HM | WT | 4.130720954 | 327.6785858 | 165.9046534 | -6.298780761 | 1.850214623 | -3.40435141 | 0.000663214 | 0.006245293 | | Down | Mylk4 |
| ENSMUSG00000037953 | HM | WT | 0.295051497 | 33.70621069 | 17.00063109 | -6.606501509 | 1.274100577 | -5.185227626 | 2.16E-07 | 7.19E-06 | | Down | A4gnt |
| ENSMUSG00000059201 | HM | WT | 0.590102993 | 75.10685819 | 37.84848059 | -6.96415471 | 2.947858542 | -2.362445352 | 0.018154818 | 0.078887547 | | Down | Lep |
| ENSMUSG00000024806 | HM | WT | 0 | 25.98213442 | 12.99106721 | -7.192544049 | 1.914293371 | -3.757284101 | 0.000171767 | 0.002081721 | | Down | Mlana |
| ENSMUSG00000033825 | HM | WT | 0.329491899 | 62.74739774 | 31.53844482 | -7.502033352 | 1.243677417 | -6.032137636 | 1.62E-09 | 9.85E-08 | | Down | Tpsb2 |
