# Supplementary material for: Full length transcriptomic profiling reveals insights into the white coat phenotype in Waardenburg syndrome mice harboring the Mitf R324del mutation
Source: Sci Rep. 2025 Jul 31;15:28012. doi: 10.1038/s41598-025-13359-8 (PMC12314077; doi:10.1038/s41598-025-13359-8)
Supplement: Supplementary file 2 — Supplementary Material 2 [file 41598_2025_13359_MOESM2_ESM.pdf]

Raw image for Figure 6B

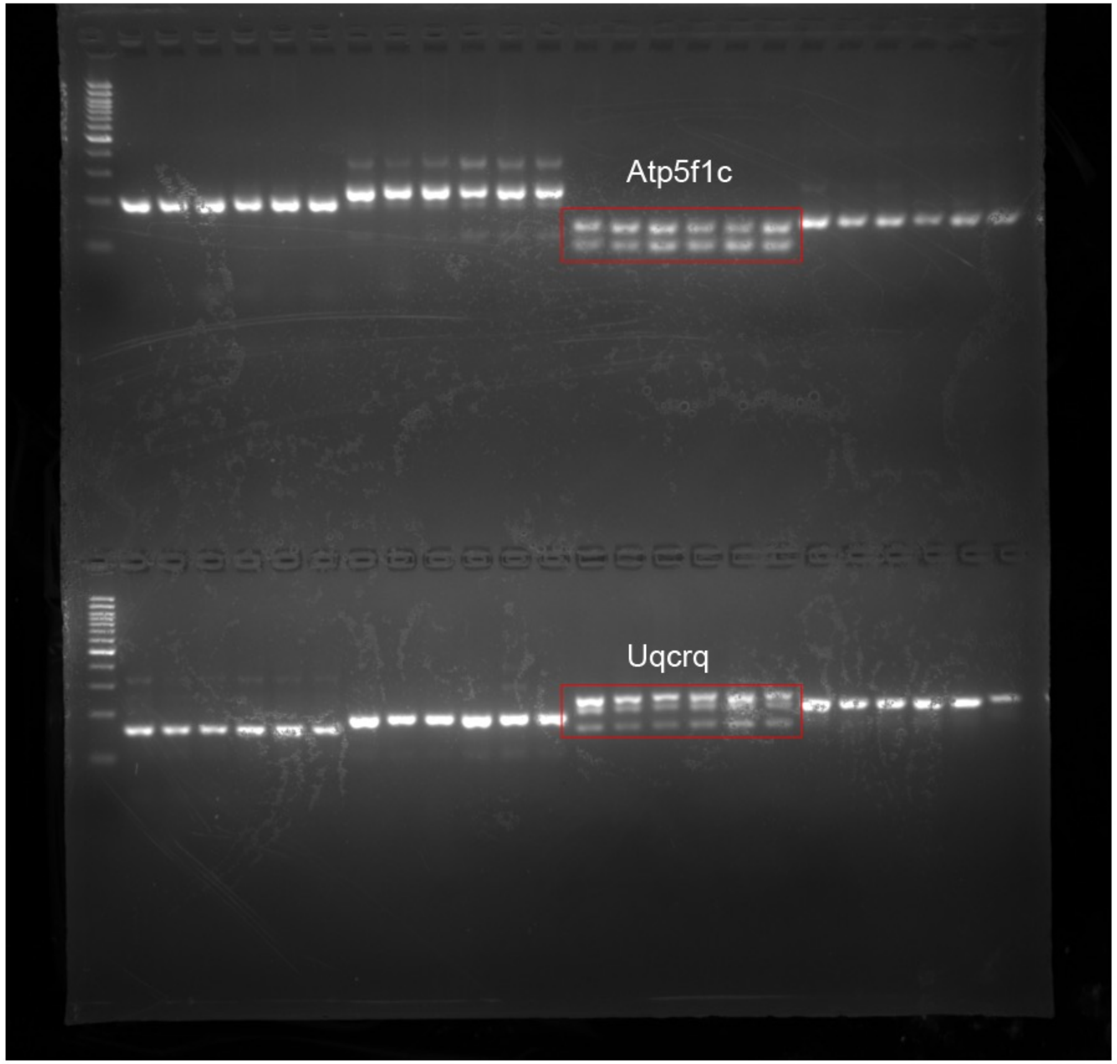

Figure 6B

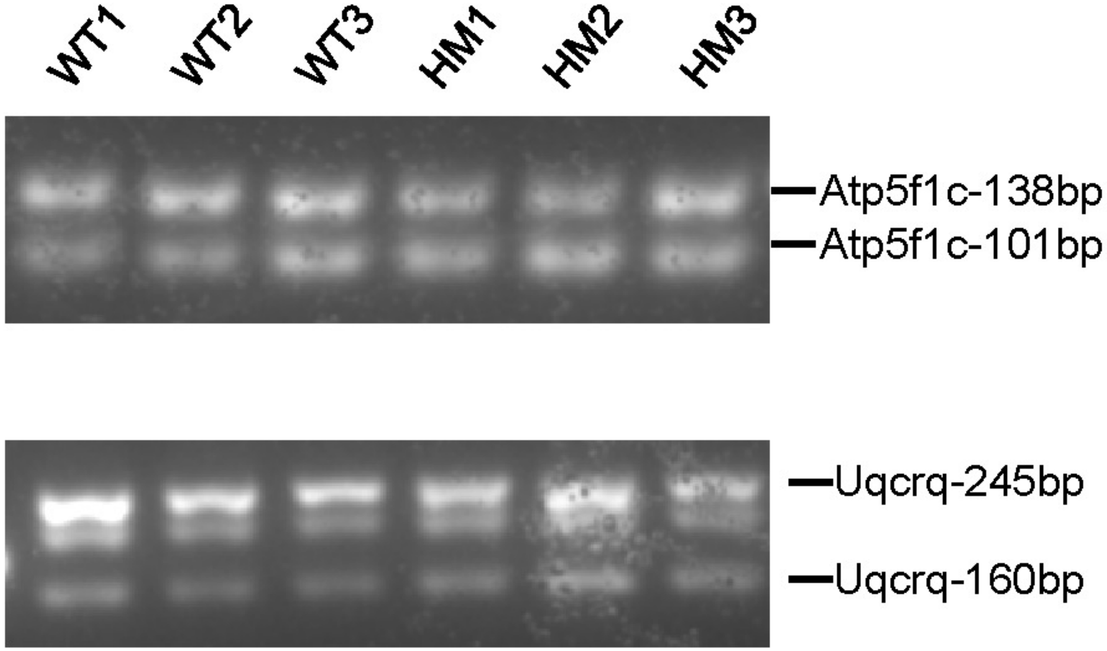

Raw image for Figure 6B (left).
Image of Figure 6B (right).
It is clear that the authors did not modify the image.
